# The role of rumen microbiota in enteric methane mitigation for sustainable ruminant production

**DOI:** 10.5713/ab.23.0301

**Published:** 2023-11-02

**Authors:** Takumi Shinkai, Shuhei Takizawa, Miho Fujimori, Makoto Mitsumori

**Affiliations:** 1 NARO Institute of Livestock and Grassland Science, Ibaraki 305-0901, Japan

**Keywords:** *Bacteriodetes*, Greenhouse Gas, Methane, *Prevotella*, Rumen, Rumen Microbiota

## Abstract

Ruminal methane production functions as the main sink for metabolic hydrogen generated through rumen fermentation and is recognized as a considerable source of greenhouse gas emissions. Methane production is a complex trait affected by dry matter intake, feed composition, rumen microbiota and their fermentation, lactation stage, host genetics, and environmental factors. Various mitigation approaches have been proposed. Because individual ruminants exhibit different methane conversion efficiencies, the microbial characteristics of low-methane-emitting animals can be essential for successful rumen manipulation and environment-friendly methane mitigation. Several bacterial species, including *Sharpea*, uncharacterized Succinivibrionaceae, and certain *Prevotella* phylotypes have been listed as key players in low-methane-emitting sheep and cows. The functional characteristics of the unclassified bacteria remain unclear, as they are yet to be cultured. Here, we review ruminal methane production and mitigation strategies, focusing on rumen fermentation and the functional role of rumen microbiota, and describe the phylogenetic and physiological characteristics of a novel *Prevotella* species recently isolated from low methane-emitting and high propionate-producing cows. This review may help to provide a better understanding of the ruminal digestion process and rumen function to identify holistic and environmentally friendly methane mitigation approaches for sustainable ruminant production.

## INTRODUCTION

Within the global food system that supports over 8 billion people, ruminant production plays an important role in meeting global food demand. However, this system produces a considerable source of greenhouse gases (GHG), accounting for 21% to 37% of total anthropogenic GHG emissions [1,2]. Livestock methane production constitutes 17% of global food system GHG emissions, of which 88% derives from enteric fermentation [3]. The growth in GHG emissions from ruminant production is estimated to increase by 8.8% during this period, and ruminants and other livestock will contribute 90% of the GHG emission increase in the agricultural sector. This is assumed to be the consequence of an expected increase in the number of dairy cows (+14%) and the global milk supply (+23%, +1.8% annually), mainly in low- to middle-income countries, and global beef and sheep production (+8% and +16%, respectively) over the next decade [4]. Based on the current situation and prospects, a transition to consumer diets containing a smaller proportion of calories from animal food sources, particularly ruminant meat, is strongly recommended to create an environmentally sustainable food system [2]. Concerted research efforts are required to develop practical items for absolute and product-based enteric methane mitigation.

In this review, we provide an overview of the extensive research into ruminal methane production and mitigation strategies, focusing on rumen fermentation and the functional role of rumen microbiota, with a special focus on the activity of *Prevotella* species. This overview may help to provide comprehensive insights into the relationship between enteric methane production and the function of rumen microbiota in methane mitigation.

### Ruminal methane production

The rumen constitutes a unique digestive system in ruminants, capable of utilizing lignocellulosic and soluble plant polysaccharides in the rumen. Rumen microbiota have coevolved with host animals to form a lignocellulosic biomass digestion system. The microbiota generate metabolic hydrogen through glycolysis, followed by acetate and butyrate production [5]. Methanogenesis occurs under strict anaerobic conditions using hydrogen, carbon dioxide, and formate, which are continuously provided during fermentation. Around 200 to 600 L of methane is produced daily by a typical dairy cows, representing a 2% to 15% loss of feed energy [6–8].

Ruminal methane production is a complex trait affected by dry matter intake (DMI) or digestible organic matter intake, feed composition, rumen microbiota, proportion of fermentation products, lactation stage, host genetics, and environmental factors [9–16]. The relationship between host animal age and age-dependent microbiota shifts must be considered [17,18]. Numerous attempts have been made to estimate ruminal methane production for inventory purposes. The amount of methane produced depends on the intake of dry or digestible organic matter [8,19], making accurate prediction of DMI (total input) essential for the accurate prediction of methane emissions (total output) [20]. Data on both of these metrics are crucial for understanding rumen fermentation and evaluating the effects of related parameters.

Propionate formation consumes either intra-(metabolic) or intercellular (di-) hydrogen. Hydrogenases may be involved for the uptake of outside hydrogen [21,22]. It is generally accepted that both methane and propionate function as the main hydrogen sinks in the rumen. These two products show a strong negative correlation, whereas high acetate and butyrate production enhances methane production by increasing hydrogen production [5,19,23]. A forage-based diet leads to a higher proportion of acetate and butyrate than a concentrate-based diet, resulting in a higher amount of methane production compared to a concentrate-based diet [23,24]. *Ruminoccoccus albus* is among the predominant fiber digesters and known to produce significant amounts of hydrogen from glucose. *R. albus* strain 7 ferments one molecule of glucose to 1.3 acetate, 0.7 ethanol, 2 CO_2_, and 2.6 hydrogen in batch culture, and to 2 acetate, 2 CO_2_, and 4H_2_ in continuous culture with hydrogen consumers [25]. Other predominant fiber digesters such as *R. flavefaciens* and *Butyrivibrio fibrisolvens* also produce significant amounts of formate and/or hydrogen [26–28]. In contrast, no hydrogen or carbon dioxide is produced by the predominant fiber digester *Fibrobacter succinogenes* during cellulose fermentation, resulting in the absence of methane production in co-culture with methanogens [29,30]. Higher propionate production has both beneficial and detrimental aspects for ruminant production. Since propionate is the most abundant precursor of glucose in host ruminants [31], higher production in the rumen may be beneficial for dairy cows with high milk production to meet their high energy requirements. However, high ruminal propionate concentrations could lead to a decrease in DMI, a phenomenon that was found to be enhanced under high ruminal ammonia concentrations [32].

Lyons et al [11] reported that methane production increased over the lactation period under constant DMI (32.2, 33.8, and 36.7 L methane per kg DMI in the early, middle, and late lactation periods, respectively). The increased methane yield in the late lactation period was reflected in changes in the microbial and archaeal community structure, featuring a significantly higher acetate and butyrate to propionate (NGR) ratio compared to the early period. The authors observed a strong positive correlation between propionate concentration and the proportional presence of the gram-negative Bacteroidetes genus *Prevotella*, suggesting a high impact of rumen microbiota and shifts in their fermentation activities on methane production.

### Methane mitigation strategies

The development of multiple enteric methane mitigation strategies is necessary to meet the needs of various farming systems and practices, consumer types, and farming environments [33]. In addition, the mitigation strategies need to maintain or increase feed utilization and animal productivity such as weight gain or milk yield. Arndt et al [3] categorized mitigation strategies into two types: product-based methane mitigation (methane per unit of meat or milk) and absolute methane mitigation. The latter is linked to the balance between generation and consumption of metabolic hydrogen in the rumen.

Beauchemin [34] suggested several concepts and a strategic planning timeline for methane mitigation in dairy cows. This encompassed various approaches including feed supplements such as oils, rumen modifiers (yeast, enzymes, direct-fed microbes), and plant extracts (tannins, saponins, oils), in addition to changes in feedstuff (diets higher in grain, legumes, corn silage, and small grain silage). Strategies related to improving feed conversion efficiency, such as animal selection and herd management to reduce animal numbers and increase milk production per cow, have also been considered. A meta-analysis summarized mitigation potentials and found that preferable methane emission intensity for weight gain and milk production was correlated with increasing feeding levels, decreasing grass maturity (increasing forage quality), using oils and fats, decreasing the forage-to-concentrate ratio, and feeding 3-nitrooxypropanol (3-NOP) and hydrogen acceptors (fumaric acid and nitrate) [3]. Although no methane emission intensity per weight gain and milk production was calculated, the methane mitigation capabilities of bromochloromethane (BCM), monensin, long-chain fatty acids, essential oils, tannins, and protozoan defaunation were also highlighted. Among these, the negative effects of condensed tannins on fiber digestibility, weight gain, and milk production must be considered. Importantly, productivity (milk production and weight gain) was unaffected by almost all examined mitigation approaches involving rumen manipulation; only nitrate feeding had a positive effect on milk production. In contrast, BCM treatments in goats, intended to drive a shift to a more propionic type of rumen fermentation, produced a 36% increase in milk yield [35]. An increase in body weight was previously reported following 3-NOP treatment in dairy cows [36].

There are limited or no viable prospects for mitigating methane emission from pastured ruminants at a global scale [33]. In particular, ruminant production in lower-middle- and low-income countries, which are expected to increase ruminant numbers and production over the next several decades, features pasture-based feeding systems that are less closely linked to commercial feed use [4]. A comparison of GHG emissions from beef and veal production by country showed that 88, 58, 35, and 16 kg of CO_2_-eq GHG were produced per 100 kg of meat production in low-, lower-middle-, upper-middle-, and high-income countries, respectively [4]. This clearly indicates that a product-based methane mitigation strategy must first be adopted in low- to lower-middle-income countries.

To evaluate the effect of reducing methane production in the rumen, a combination of BCM and α-cyclodextrin (CD) can be used [35,37,38]. Methanogens utilize methyl coenzyme M reductase in the final step of methanogenesis. BCM is believed to inhibit methane production by reacting with reduced vitamin B12 and inhibiting cobamide-dependent methyltransferase, which affects coenzyme M synthesis [38, 39]. BCM is highly volatile but chemically stable in combination with CD [38]. Use of BCM-CD feeding successfully inhibited over 90% of methane production in goats [37] and demonstrated no effect on feed intake or digestibility of dry matter, organic dry matter, or neutral detergent fiber. This observation was supported by the quantitative analysis of fiber digesters; the relative abundance of hydrogen- and formate-producing fiber digesters such as *R. flavefaciens* and fungi decreased [26], whereas that of non-hydrogen-producing *F. succinogenes* slightly increased in BCM-CD-treated rumen microbiota. Similar digestibility results were also reported in a batch and continuous culture study under 85% to 90% methane inhibition [40]. The polysaccharide digestibility of *R. flavefaciens* decreased in the absence of methanogens, whereas that of *F. succinogenes* was not affected [41]. Thus, the increase in *Fibrobacter* seems to compensate for the decrease in *R. flavefaciens* and fungi to maintain fiber digestibility in high methane-mitigating conditions [37]. Under such conditions, propionate concentration and the acetate-to-propionate ratio increased [37]. Improvements in the acetate-to-propionate ratio and feed efficiency have been reported in steers under long-term BCM feeding [38], and a 36% increase in goat milk production was observed alongside a 33% methane mitigation under BCM use [35]. However, multiple further nutritional evaluations are necessary because the rumen microbiome structure and fermentation pathways were drastically altered in BCM-treated goats [37,42].

Ciliate protozoa, generally present in concentrations of 10^4^ to 10^6^ individuals per gram, can produce hydrogen via hydrogenosomes [43,44]. Some methanogens closely associate with protozoan populations on their exterior surfaces and/or present as endosymbionts to increase hydrogen availability [45–47]. These associations consist of protozoa-archaea specific attachments through adhesin-like proteins [48]. Possible methane production related to protozoan populations was estimated at up to 35% in sheep [49,50] and 9% to 25% and/or 37% *in vitro* [45,51]. Guyader et al [52] found that in almost all lipids feeding trials resulted in a concomitant mitigation of both protozoan concentration and methane emission. Although defaunation techniques require further assessment for routine use in farms, defaunation or maintaining lower protozoan populations with lipid feeding may need to be considered as a potential methane mitigation option.

Various types of methane-mitigating agents, including 3-NOP, *Asparagopsis taxiformis*, monensin, cashew nutshell liquid (CNSL), and nitrate, have been tested. No apparent toxicity symptoms were observed for these compounds [53–55]. Continuous efforts to identify new inhibitors of ruminal methane mitigation are required because rumen microbiota have adapted to various types of phytotoxins and antimicrobial plant materials taken into the rumen by the host ruminant; multiple inhibitors may thus be necessary for long-term methane suppression, probably involving alternate use. Weimar et al [56] developed a high-throughput screening method using a multiwell plate for strictly anaerobic microorganisms and screened 120 active compounds from 1,280 compounds listed in the Sigma-Aldrich LOPAC compound library. This screening method is expected to facilitate further investigation of many other compound libraries. To achieve sustainability, long-term influences on cow health, propagation, chemical breakdown, and accumulation in the environment need to be considered, especially for halogenated compounds, including bromoforms, involved in *Asparagopsis taxiformis* supplementation [54,57,58].

### Rumen microbiota in lower-methane emitters

Rumen microbiota are an important factor affecting low methane production and high feed efficiency. In general, rumen microbiota are dominated by a core of poorly characterized microbes [59,60]. A worldwide survey found that microbial community composition was predominantly attributed to diet, but core microbes were geographically distributed irrespective of diet [60]. Individual ruminants exhibit different methane conversion efficiencies (methane production per unit of DMI) under the same feeding and environmental conditions. Since rumen microbiota differ between high- and low-methane-producing ruminants, understanding the characteristics of rumen fermentation and rumen microbiota in lower methane-emitting ruminants is important. Heritable core rumen microbes may be the primary targets for rumen manipulation for environmentally friendly methane mitigation [13].

Microbes are present in the rumen in three interconnect ed environments. The solid phase, liquid phase, and surface of rumen epithelium and protozoa respectively hold 70%, 25%, and 5% of microbial biomass in the rumen [61]. In most rumen microbiome analyses, the liquid phase has been used due to the technical issue of sampling. By comparing liquid phase samples, the abundance of several bacterial species differs between lower and higher methane emitters.

Basic information is available to support an understand ing of the specific relationship between bacteria and archaea and the shift in rumen microbiota under conditions of high methane mitigation. A worldwide survey found no strong association between the most abundant bacteria and archaea [60]. However, distinct positive and specific relationships were detected between less abundant bacteria, including succinate-producing Succinivibrionaceae, succinate-utilizing *Dialister*, amino acid-utilizing *Acidaminococcus*, and archaea, including *Methanomassiliicoccaceae*, *Methanosphaera*, and *Methanobrevibacter boviskoreani* [60]. Another positive relationship was detected between *Lachnospiraceae* and the methylotrophic methanogen *Methanosphaera* [60]. These specific and positive relationships are considered to be related to methanol production and utilization.

In studies involving low methane-emitting animals, a re lationship between enteric methane production and an uncharacterized Succinivibrionaceae, as well as *Prevotella* sp., has been suggested in both beef and dairy cows [10,15,62]. The family Succinivibrionaceae includes *S. dextrinosolvens*, *Ruminobacter amylophyllus*, and a phylogenetically different Tammar wallaby isolate that produces succinate and acetate [63]. Another uncharacterized Succinivibrionaceae related to low methane production has yet to be cultured. Although the phylogenetic information is quite limited, taxa in the family Succinivibrionaeae were detected more frequently (by ~10%) in low methane emitting Aberdeen-Angus or Limousin crossbreed steers fed concentrate-based diet [62]. A specific operational taxonomic unit (OTU) assigned to unclassified Succinivibrioneceae was detected in Holstein-Friesian bulls fed concentrated diets with high feed efficiency, but decreased under feed-restricted conditions [64]. Similar observations were made in Swedish Red and Holstein dairy cows, where a total 7% of several *Prevotella* OTUs and 2% in the family Succinivibrionaeae were detected as low methane-emitting cow-characteristic bacteria in the mid-lactation period [15]. In contrast, these unclassified Succinivibrioneceae were not highlighted in another study on high feed efficiency and low methane production in Holstein Friesian dairy cows fed concentrate-based diets [65]. In that study, sequenced genomes related to the acrylate pathway and assigned to lactate-using *Megasphaera elsdenii* and *Coprococcus catus* (Lachnospiraceae) were found to be enriched in efficient cows. The functional characteristics of most of the listed bacteria remain unclear, as they are yet to be cultured. Importantly, this indicates that unclassified Succinivibrionaceae appeared to be enriched in ruminants fed a sufficiently concentrated diet. Wallace et al [62] pointed out the effect of low-pH conditions on the relationship between low methane emissions and rumen microbiota. In fact, ruminal methanogenesis is highly sensitive to low pH [66], whereas *Megasphaera* can survive under low-pH ruminal acidosis conditions [67].

In the rumen of sheep, three different microbiota named ‘ruminotype’ associated with methane production were identified [14]. Of these, two showed lower methane production, one with a lower acetate-to-propionate ratio (ruminotype Q) and one without an SCFAs profile (ruminotype S). Ruminotype Q had a higher proportion of propionate-producing *Quinella ovalis*, while ruminotype S had a higher proportion of lactate- and succinate-producing *Sharpea azabuensis*, *Fibrobacter* spp., *Kandleria vitulina*, *Olsenella* spp., and *P. bryantii*. The rumen microbiome of ruminotype S features rapid heterofermentative fermentation induced by *Sharpea*, leading to lactate production [68]. It is thought to convert lactate to butyrate, mainly via *Megasphaera*, although conversion to propionate is preferred for methane mitigation and feed efficiency. Lower methane production under the two-step fermentation process via lactate to butyrate was attributable to lower hydrogen production.

Age-dependent shifts in microbial associations have also been reported. Liu et al [18] found a strong correlation between *Prevotella* and *Methanobrevibacter* in younger heifers, which was replaced by a correlation between *Succinivibrio* and *Methanobrevibacter* in older cows. Microbial diversity decreases age-dependently in primiparous and multiparous cows [17]. In addition, based on rumen microbiome analysis, a *Sharphea*-enriched community shift was proposed to be caused by physical differences in rumen size and turnover rate [68]. Because the association between rumen microbiota and host age, host genetics, rumen size, feed passage, digestive rate, and rumen acidity is still unclear, future collaborative research into these aspects is necessary [69].

### Hydrogen uptake associated with utilization of fermentation intermediates

Non-volatile organic acids generated by rumen bacteria, including succinate, malate, fumarate, and lactate, are immediately secondarily fermented as intermediate metabolites during rumen fermentation. The production and utilization of these nonvolatile organic acids seem to play an important role in rumen microbiota. Several non-volatile organic acid-utilizing bacteria, including *M. elsdenii*, *Dialister* sp., and selenomonads, have been found to be characteristic to low methane-emitting animal [9,62,68]. *Dialister* was found in low methane-emitting cows, even though a worldwide survey showed a positive and specific relationship between methanogens [60]. *Bacteroidetes* and *Wolinella succinogenes* can utilize hydrogen [70,71], and the NiFe-hydrogenase-encoding genes present in *W. succinogenes* have also been found in several Proteobacteria strains [71]. Greening et al [9] identified 26 distinct hydrogenase subgroups and gene expressions of various hydrogen uptake pathways in the rumen microbiome of sheep. The study suggested methane mitigation strategies using complex hydrogen fluxes, including fumarate, nitrate, and sulfate respiration. Several non-volatile organic acids are promising hydrogen acceptors for propionate production. *P. ruminicola*, *Anaerovibrio lipolytica*, and *Selenomonas ruminantium* can use membrane-bound hydrogenases and extracellular hydrogen to reduce fumarate to succinate [72]. In addition, a certain amount of methane can be mitigated by feeding propionate precursors such as fumarate or malate. In a pilot study, 1% of fumarate feeding per dry matter (DM) did not affect methane production in Angus heifers [73]. In that study, the fumarate feeding level was minimized to neutralize the acidity of the fumaric acid treatment. Wood et al [74] succeeded in feeding 10% of fumarate in the diet using a capsulation technique, and successfully decreased methane production in growing lambs by 76%. This inhibition rate was much higher than the of 20% reduction observed in a previous *in vitro* experiment. An addition of 7.5% of malate feed per DM also decreased methane production by 9% per unit of DMI [75]. However, the affinity of fumarate-utilizing and hydrogen-consuming bacteria for hydrogen is expected to be lower than that of methanogens [76]. To enhance bacterial hydrogen uptake, hydrogen-utilizing and non-volatile organic acid utilizers in the rumen should be the focus of research, despite their lower proportions in the rumen microbiome.

Three pathways (succinate, acrylate, and propanediol for propionate production) consume hydrogen in the microbiome both of the human gut and the rumen [22,65]. The composition of lactate-utilizing bacteria may vary as lactate permease genes are found in various gut bacteria, including the genera *Bacteroides*, *Prevotella*, *Bacillus*, *Lactobacillus*, *Clostridium*, *Intestinibacter*, *Coprococcus*, *Eubacterium*, *Megasphaera*, *Mitsuokella*, *Selenomonas* and *Veillonella* [77]. The well-known lactate-utilizing *M. elsdenii* can convert lactate to mainly acetate and butyrate (42% and 30%, respectively) with lesser proportions of propionate (28%) [78]. Another study reported that the lactate was converted into acetate (43%) and propionate (53%) under batch conditions, whereas primary butyrate (no propionate) conversion occurred without acrylate supplementation under carbon-limited steady-state conditions [79]. This is because *M. elsdenii* oxidizes lactate to acetate to generate ATP and simultaneously reduces lactate to propionate via the acrylate pathway [79]. Methane production increased when *M. elsdenii* was added to rumen fluid obtained from goats but decreased with the addition of *S. ruminantium* subsp. *lactilytica* under *in vitro* conditions [80]. This result was confirmed by co-culture trials with methanogens and bacteria in lactate cultures [80]. Thus, the efficient conversion of lactate to propionate is important for further hydrogen uptake and methane mitigation.

### The importance of functional diversity within the genus *Prevotella*

The genus *Prevotella* consists of more than 60 species with high phenotypic, genetic, and ecological diversity [81,82]. Based on a 16S rRNA gene-based phylogenetic tree, the ruminal *Prevotella* species *P. ruminicola*, *P. albensis*, *P. brevis*, and *P. bryantii* form separate clusters against other ecologically different *Prevotella* species [83,84]. There are reports of not only phylogenetic diversity but also genetic differences, including the carbohydrate-active enzyme (CAZyme) profile [84,85]. Emerson and Weimer [27] compared the fermentation products of four known ruminal *Prevotella* species and found significant differences in their end products, including a CO_2_ fixation ability in *P. bryantii*. Similarly, although *P. ruminicola* is known to take up hydrogen to reduce fumarate to succinate [72], it remains unclear whether this important ability for methane mitigation is shared beyond *Prevotella* species. Discovery of *Prevotella* clones specific to hay or concentrate diets implies functional diversity, which may partially reflect genetic differences within the genus [86]. The genus makes up 20% to 60% of total rumen bacteria by abundance, and most of *Prevotella* clones showed <97% sequence similarity with known rumen strains [86,87]. Based on these genetic and functional differences among *Prevotella* species, the importance of understanding the role of different biotypes of the genus on methanogenesis and feed efficiency has been emphasized, mainly because the abundance of certain *Prevotella* phylotypes increases or decreases according to methane production and/or feed efficiency [10,15,88–90]. We recently isolated a novel *Prevotella* species, *P. lacticifex*, from the rumen of cows with low methane and high propionate production [83]. This bacterium is unique in its higher lactate production compared to other *Prevotella* species, a functional trait similar to that of *Sharpea* in low-methane-emitting sheep [14,68]. It currently appears that isolation and culturing are the only reliable methods to reveal the physiological characteristics of bacteria and provide strong microbiological reference material. Several other uncultured *Prevotella* species are awaiting isolation for further understanding of their physiological characteristics.

Another approach was proposed to classify *Prevotella* species and/or strains into seven clades and separate them into multiple genera based on average amino acid identity (AAI), protein family (Pfam) profiles, CAZymes, marker gene sets, etc. [91]. The distribution of niches was found to be unique for each species but inconsistent within clades. The bovine rumen microbiota mainly contain the clades labeled “2” (including *P. multisaccharivorax* [proposed to be renamed to ‘*Hallella*’]), “3” (including *P. bryanttii*, *P. albensis*, and *P. copri* [proposed to be renamed to ‘*Segatella*’]), and “6” (including *P. ruminicola* and *P. brevis* [proposed to be renamed to ‘*Xylanibacter*’]). These genetic and semi-functional classification approaches are expected to facilitate the functional estimation of uncultured *Prevotella* species. Culture-independent single-cell amplified genome analysis may be another approach to generate genomic reference material for uncultured rumen bacteria [92–95].

## IMPLICATIONS

To achieve a sustainable level of methane production from this livestock, enteric methane mitigation strategies that satisfy various farming systems and practices, consumer types, and farming environments are required. Among the various mitigation approaches, microbial additives and/or electron acceptors could be the primary targets for rumen manipulation for environmentally friendly methane mitigation. Bacteria in the genus *Prevotella*, particularly *P. lacticifex*, have the potential to be used as a microbial additive to enhance propionate production and consequently methane mitigation. Further research into this and other promising mitigation methods will be crucial for achieving a holistic and environmentally friendly approach to sustainable ruminant production.
